# Hemophagocytic Lymphohistiocytosis in a Patient With Hodgkin lymphoma and Concurrent EBV, CMV, and *Candida* Infections

**DOI:** 10.1177/2324709616684514

**Published:** 2017-01-01

**Authors:** Moaath Mustafa Ali, Ana Lucia Ruano Mendez, Hetty E. Carraway

**Affiliations:** 1 Cleveland Clinic, Cleveland, OH, USA

**Keywords:** hemophagocytic lymphohistiocytosis, Hodgkin lymphoma, Epstein-Barr virus, cytomegalovirus, *Candida*

## Abstract

Hemophagocytic lymphohistiocytosis (HLH) is a syndrome characterized by immune activation and subsequent widespread organ damage. Patients affected by HLH commonly develop fever, cytopenias, liver damage, neurologic manifestations, and hypercytokinemia. In this case, we describe a 60-year-old male who presented with HLH and concurrent Epstein-Barr virus, cytomegalovirus, and *Candida* infections and was subsequently diagnosed with a Hodgkin lymphoma. This case highlights the importance of considering a cancer diagnosis in the differential diagnosis of patients presenting with HLH.

## Introduction

Hemophagocytic lymphohistiocytosis (HLH) is a disease that results from severe unchecked immune activation; it is associated with multi-organ damage and high morbidity and mortality. It is a disorder that mainly affects infants and children and less commonly adults, and it has a slight male preponderance.^1^ This disease is classified as primary HLH when the presence of an inherited genetic abnormality is identified or secondary HLH when the disease is triggered by an inflammatory process from an infectious, rheumatologic, or neoplastic disorder.^2^ The diagnosis of HLH requires a combination of clinical, laboratory, and histopathologic findings. Table 1 demonstrates the diagnostic criteria for HLH proposed by the Histiocyte Society in 2004.^3^

**Table 1.** Diagnostic Criteria for Hemophagocytic Lymphohistiocytosis Used in the HLH-2004 Trial.^a^

Molecular diagnosis consistent with HLH:
● Pathological mutations of *PRF1, UNC13D, STXBP1, RAB27A, STX11, SH2D1A*, or *XIAP*
OR
Five of the following criteria:
● Fever of 38.5°C or more
● Splenomegaly
● Cytopenias (affecting at least 2 of 3 cell lineages in the peripheral blood)
○ Hemoglobin <9 g/dL (for infants <4 weeks, hemoglobin <10 g/dL)
○ Platelets <100 000/µL
○ Absolute neutrophil count <1000/µL
● Hypertriglyceridemia (fasting triglycerides >265 mg/dL) and/or hypofibrinogenemia (fibrinogen <150 mg/dL)
● Hemophagocytosis in bone marrow, spleen, lymph nodes, or liver
● Low or absent natural killer–cell activity
● Ferritin greater than 500 ng/mL
● Increased soluble CD25 concentration (alpha chain of soluble interleukin-2 receptor)

Abbreviation: HLH, hemophagocytic lymphohistiocytosis.

a See Henter et al.^3^

In this case, we describe the diagnosis and management of a 60-year-old male who presented with multiple concurrent infections including Epstein-Barr virus (EBV), cytomegalovirus (CMV), and *Candida* infections with his initial presentation of HLH, and he was subsequently diagnosed with Hodgkin lymphoma.

## Case Report

A 60-year-old male with a past medical history of hypertension, aortic stenosis, and coronary artery disease presented initially to an outside hospital with a 6-week history of fatigue and daily fevers, with a maximum temperature up to 39.4°C. The fevers were associated with headache and blurred vision without focal neurologic deficits or nuchal rigidity. His review of systems was notable for diaphoresis, 20-pound weight loss, bilateral leg swelling, new-onset jaundice, and increasing dyspnea. On physical exam his vital signs revealed a blood pressure of 118/70 mm Hg, pulse 80 bpm, respiratory rate 15 breaths/min, and temperature 39.4°C. He was jaundiced and had hepatosplenomegaly, bilateral leg edema, and a normal neurologic exam. On laboratory examination his red blood cell count was 2 460 000/mL, hemoglobin 6.9 g/dL, white blood cell count 6900/µL, C-reactive protein 40 mg/dL, and alkaline phosphatase 418 U/L. Further laboratory testing was notably negative for parvovirus, Lyme disease, and viral hepatitis panel. Bone marrow aspirate and biopsy showed hemophagocytosis. The patient was transferred to Cleveland Clinic for further evaluation and management.

**Figure 1.**
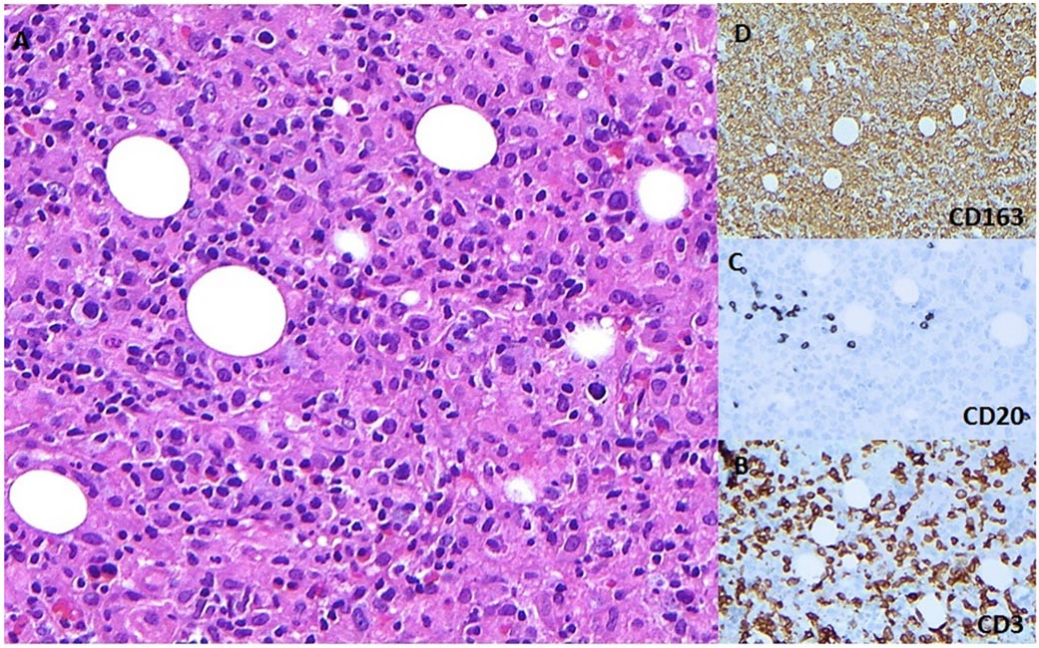
Bone marrow biopsy showing aggregates composed of small, mature lymphocytes admixed with numerous histiocytes (A) and immunohistochemical stains for CD3 (B), CD20 (C), and CD163 (D) highlighting a predominance of T-cells within these aggregates as well as the numerous histiocytes (A-C: ×200; D: ×100 magnification).

At Cleveland Clinic, a repeat bone marrow biopsy was performed since the outside hospital results were not immediately available, and this showed an increase in histiocytes with hemophagocytosis. CMV immunostain, Epstein-Barr encoding region (EBER) in situ hybridization stain, and lymphoma infiltrations were negative (Figure 1). Based on the clinical presentation, laboratory examination results, and bone marrow biopsy, he was diagnosed with HLH and was transferred to the inpatient acute leukemia service. Table 2 demonstrates laboratory workup at presentation to our facility. Notably, the patient tested positive for CMV and EBV viremia (Table 2), and the infectious disease service was consulted and the decision was made to monitor closely and follow clinically.

**Table 2.** Laboratory Data at the Time of Diagnosis.

Parameter (Normal Range)	Value at HLH Diagnosis (July 18-22, 2015)	Value at Hodgkin Lymphoma Diagnosis (August 6, 2015)
Leukocyte count (3.7-11 k/µL)	2.61	0.32
Neutrophils (1.45-7.5 k/µL)	1.83	Too few cells
Lymphocytes (1-4 k/µL)	0.52	Too few cells
Hemoglobin (13-17 g/dL)	8.7	8.3
Platelets (150-400 k/µL)	22	22
Ferritin (18-399 ng/mL)	8270	NA
Triglycerides (30-149 mg/dL)	276	NA
sCD25 (≤1033 pg/mL)	81 100	NA
LDH (100-220 U/L)	677	NA
Serum sodium (135-146 mmol/L)	140	134
AST (7-40 UI/L)	166	38
ALT (5-50 UI/L)	62	106
Total bilirubin (0.0-1.5 mg/dL)	10.1	7.3
Bilirubin, direct (0.0-0.4 mg/dL)	8.9	NA
GGT (0-50 U/L)	321	NA
AP (40-150 U/L)	423	185
BUN (10-25 mg/dL)	20	30
Creatinine (0.7-1.4 mg/dL)	1.03	0.71
Prothrombin time (8.4-13 seconds)	12.6	11
Fibrinogen (200-400 mg/dL)	109	240
Hemophagocytosis feature	Bone marrow, liver	NA
EBV DNA, quantitative (negative copies/mL)	183 912	118 665^a^
EBV VCA, IgM (<0.9 AI)	<0.2	NA
CMV DNA (negative IU/ mL)	1400	1020

Abbreviations: HLH, hemophagocytic lymphohistiocytosis; sCD25, soluble CD25; NA, not available; LDH, lactate dehydrogenase; AST, aspartate transaminase; ALT, alanine transaminase; GGT, γ-glutamyl transferase; AP, alkaline phosphatase, BUN, blood urea nitrogen; EBV, Epstein-Barr virus; CMV, cytomegalovirus.

a EBV titer on July 28, 2015.

Further analysis of bone marrow showed a karyotype of 94,XXYY,add(1)(p36.3)x2,der(1)t(1;6)(p10;p10),add(7)(q22),+8,+9,add(16)(p13.3)x2,+ mar[cp2]/46,XY[18]. These abnormalities were described as “not characteristic of a specific disease type; however, presence of an abnormal clone was highly concerning for a neoplastic disorder. The abnormalities suggested lymphoid origin.”

**Figure 2.**
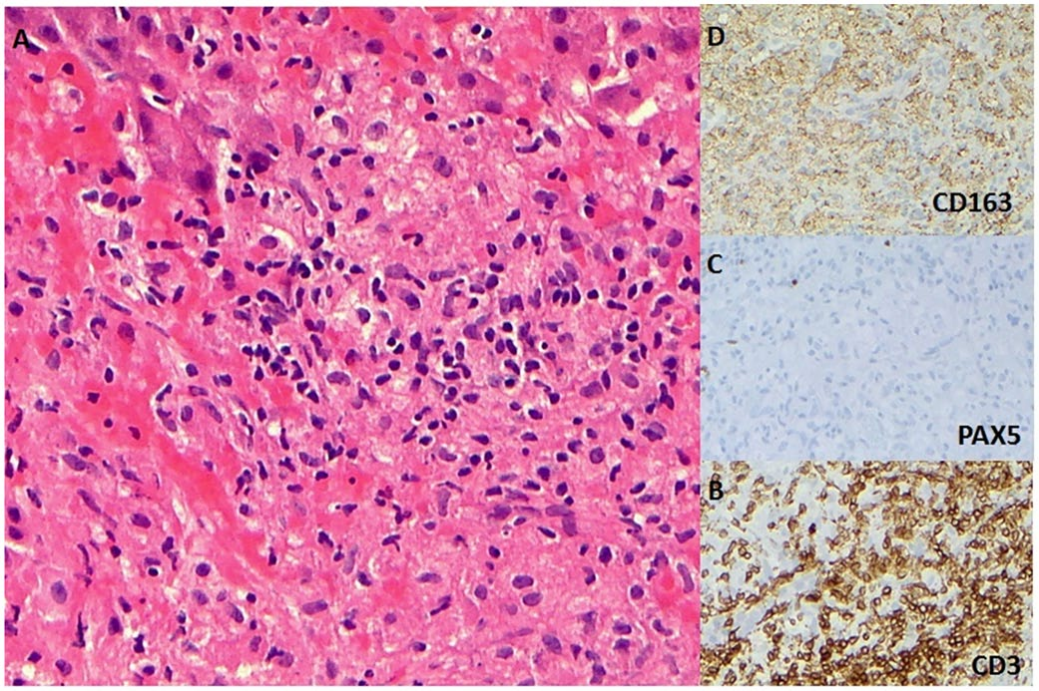
Liver biopsy images (×200) showing a lymphohistiocytic infiltrate involving the liver parenchyma (A) with a predominance of T-cells highlighted by CD3 immunohistochemical stain (B), rare B-cells as shown by PAX-5 (C), and a proliferation of histiocytes highlighted by CD163 (D).

The patient continued to have fever, fatigue, and increasing liver enzymes. Despite repeat blood cultures and urine cultures, there was no detectable bacterial infection. He was treated with several antibiotic courses during his initial neutropenia. Computerized tomography of the chest and abdomen revealed trace left and right pleural effusions, bronchocentric macronodular opacities in bilateral upper lobes, multifocal hypodense lesions in the liver and spleen, and enlarged periportal (largest: 5.2 × 2.1 cm) and peripancreatic lymph nodes. Magnetic resonance imaging with contrast was done; however, it was indeterminate. The focal liver lesion was biopsied and revealed a lymphohistiocytic infiltrate consistent with HLH (Figure 2).

Because of the patient’s systemic inflammatory response syndrome and the progressive increases in liver enzymes and bilirubin, treatment for HLH was initiated with dexamethasone 23 mg intravenous (IV) daily and etoposide (VP-16) 75 mg/m^2^ (171 mg) twice weekly. He also received 2 doses of 80 mg intravenous immunoglobulin. After improvement of the hepatic enzymes and bilirubin, the VP-16 dose was increased to 112.5 mg/m^2^ (243 mg).

**Figure 3.**
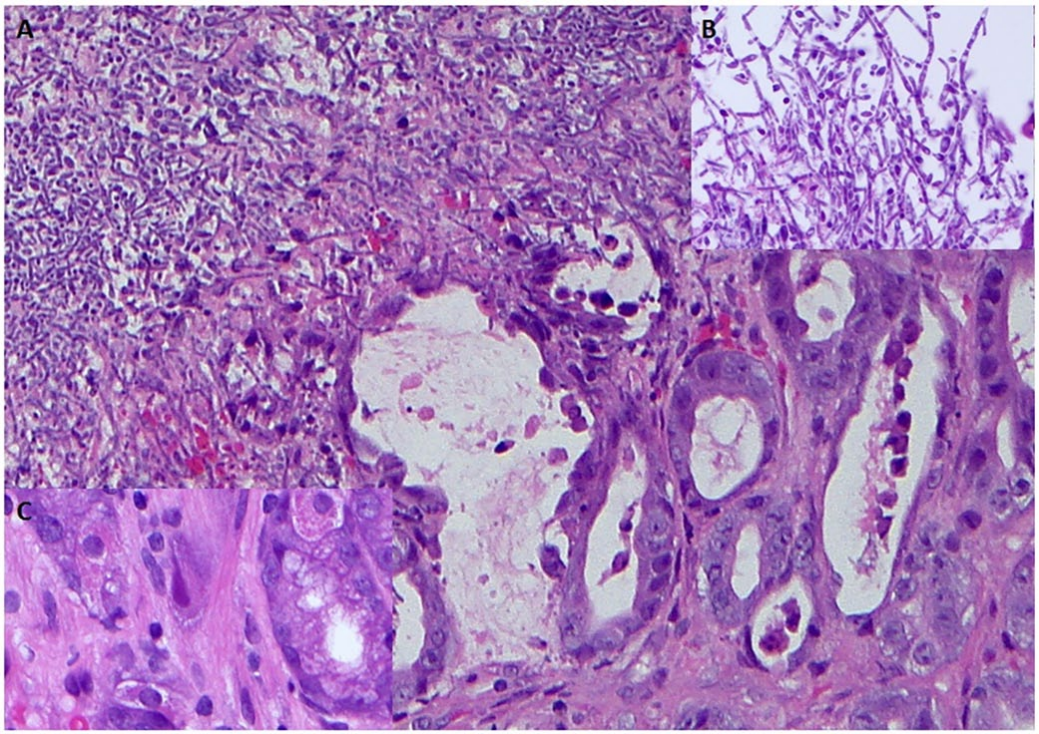
Gastric biopsies showing areas of ulceration with reactive epithelial changes and numerous fungal organisms morphologically consistent with *Candida* spp (A: ×200, B: ×500 magnification). Stromal cells containing viral inclusions, consistent with cytomegalovirus infection, were also identified (C: ×400).

He clinically improved with resolution of fever, fatigue, and abnormal liver function tests. In order to maximize the biopsy yield to exclude lymphoma, VP-16 was stopped after a total number of 3 doses. Biopsy of the periportal lymph node through upper endoscopic ultrasound guidance was attempted, at which time gastric ulcers were discovered and biopsied. The procedure was terminated due to excessive bleeding post gastric biopsy. Laparoscopic lymph node biopsy was performed without complications. The gastric biopsy was consistent with active gastritis with CMV inclusions and fungal forms morphologically consistent with *Candida* species; it was negative for chromogenic in situ hybridization of EBV (Figure 3). The patient was initiated in ganciclovir 420 mg IV BID and fluconazole 400 mg daily for 5 days.

**Figure 4.**
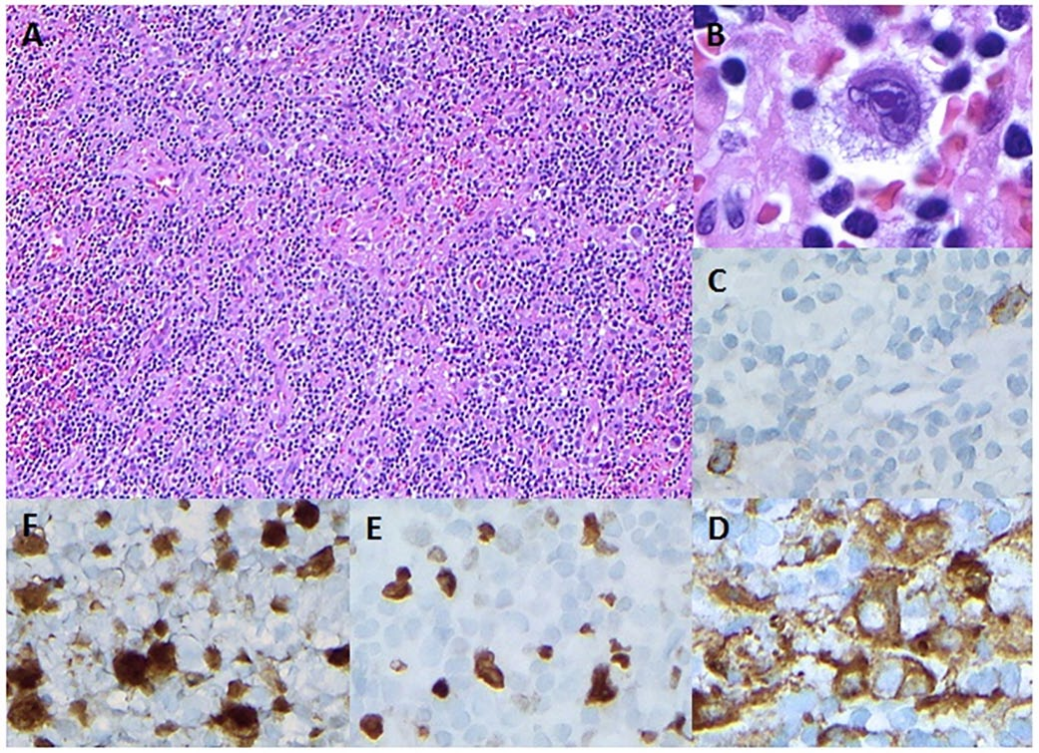
Periportal lymph node biopsy showing diffuse lymphohistiocytic infiltrates (A: ×200 magnification) with few, scattered atypical cells showing irregular nuclear contours and prominent eosinophilic nucleoli (B: ×1000). Immunohistochemical stains show that the atypical cells are positive for CD30 (C), fascin (D), MUM1 (E), and BOB1 (F) (C-F: ×400 magnification).

The lymph node biopsy diagnosed mixed cellularity Hodgkin’s lymphoma and was negative for EBER in situ hybridization (Figure 4). He received one dose of rituximab 780 mg IV and began standard therapy with the ABVD chemotherapy regimen (doxorubicin, bleomycin, vinblastine, and dacarbazine). In order to reduce bilirubin and subsequently initiate ABVD, the patient was started on dexamethasone 40 mg by mouth daily for 4 days and cyclophosphamide 750 mg/m^2^ intravenously once with 2-mercaptoethane sulfonate sodium (mesna; which reduces the incidence of hemorrhagic cystitis when cyclophosphamide is given). However, his bilirubin continued to increase, so he was treated with 2 external beam radiotherapy sessions to the liver, and his bilirubin subsequently decreased. Repeat EBV titers were negative. He was subsequently started on modified ABVD (omitting bleomycin). Repeat CMV titers were negative, but the patient continued ganciclovir to complete a total of 3 weeks of therapy. The patient improved clinically; his complete blood counts, liver enzymes, and bilirubin all improved. However, he continued to have febrile episodes and thus had several blood cultures, a transthoracic echocardiogram, and extensive fungal workup, all of which were unrevealing. Head, neck, chest, and abdomen computed tomography scans were also negative. Broad-spectrum antibiotics were stopped and patient remained hemodynamically stable, and it was thought his fever was from HLH. He was discharged on August 31, 2015, with an appointment with the lymphoma clinic on September 4, 2015.

## Discussion

In this case, we present a patient with secondary HLH in the setting of Hodgkin’s lymphoma and a picture of immune dysregulation; our patient had features of immune activation (ie, fatigue, fever, and increased inflammatory markers) as well as immune deficiency (ie, opportunistic CMV, EBV, and *Candida* infections). Defective granule-mediated cytotoxicity is considered to be the main pathophysiology in primary and secondary HLH.^4^ Perforin and *Fas* play an important role in T-cell and dendritic homeostasis, and loss of their function might lead to unchecked inflammation.^5^ The defect in these systems might also explain the strong association described between HLH and opportunistic infections.^6-8^ Perforin gene knockout mice demonstrate immunodeficiency and a disease similar to HLH when infected with lymphocytic choriomeningits virus.^9,10^ Moreover, there is an increased incidence of lymphoproliferative diseases in these mice, especially in the setting of concurrent *Fas* gene mutation.^9,11^ Clementi et al demonstrated that in 29 patients with lymphoma and clinical or pathologic hemophagocytosis or liver disease, 4 patients were homozygous for perforin mutation.^12^ *Fas* somatic mutation was also described in lymphoproliferative diseases.^13^ The loss of function of those genes may lead to defective immune regulation and increased risk of infections, malignancy, and dysfunctional immune activation. Ménard et al demonstrated that among 34 patients with Hodgkin’s lymphoma and HLH, 32 had positive EBV detected in tumor cells.^14^

The association between HLH and hematologic malignancies including Hodgkin’s lymphoma is well described, with 1% of these patients developing HLH.^15^ Tumors can cause HLH by excessive secretion of cytokines.^16^ In this case, HLH was likely triggered by the tumor and infectious agents: CMV infection, EBV infection, and/or *Candida* infection. This indicates that patients with HLH can have several concurrent diseases and that further search for underlying triggers and predisposing factors should be pursued. In this patient, a lymph node biopsy led to a more definitive answer. This case highlights the importance of looking for underlying malignancy in adults presenting with HLH. Interestingly, this patient’s EBV and CMV viremia failed to resolve—even with antiviral therapy—except until starting definitive lymphoma treatment with infliximab and modified ABVD.

In conclusion, we present a patient with HLH and multiple opportunistic infections in the setting of Hodgkin’s lymphoma. Comprehensive testing and accurate diagnosis are important strategies for successful treatment of patients with HLH. Pathophysiology of HLH remains obscure. Secondary HLH may have underlying genetic mutations explaining the association between HLH and other disease; however, further studies are needed to explore this association.
